# Editorial: the 15th annual *Nucleic Acids Research* Web Server issue 2017

**DOI:** 10.1093/nar/gkx457

**Published:** 2017-06-21

The 2017 Web Server issue of *Nucleic Acids Research* is the 15th in a series of annual issues dedicated to web-based software resources for analysis and visualization of molecular biology data. It is freely available online under *NAR*'s open access policy. This year, 284 proposals were submitted and 102, or 36%, were approved for manuscript submission. Of those approved, 86, or 84%, were ultimately accepted for publication. Table 1 lists the 2017 Web Servers, their URLs, and a brief description of each.

**Table 1.** Descriptions of web servers in the *NAR* 2017 Web Server issue

**Web Server name**	**URL**	**Brief description**
**agriGO v2**	http://systemsbiology.cau.edu.cn/agriGOv2/	GO analysis for agricultural species
**AMMOS2**	http://drugmod.rpbs.univ-paris-diderot.fr/ammosHome.php	Energy minimization of protein–ligand complexes
**antiSMASH**	http://antismash.secondarymetabolites.org/	Secondary metabolite biosynthetic gene cluster mining in bacterial and fungal genomes
**ARTS**	http://arts.ziemertlab.com	Biosynthetic gene cluster mining for novel antibiotics
**BAR 3.0**	http://bar.biocomp.unibo.it/bar3	Protein structure and function annotation
**BepiPred-2.0**	http://www.cbs.dtu.dk/services/BepiPred-2.0/	B-cell epitope prediction from a protein sequence
**BioAtlas**	http://bioatlas.compbio.sdu.dk	Visualization of microbiome and metagenome locations
**BIS2Analyzer**	http://www.lcqb.upmc.fr/BIS2Analyzer/	Analysis of coevolving amino-acid pairs in protein sequences
**BusyBee**	https://ccb-microbe.cs.uni-saarland.de/busybee	Metagenome binning
**CAFE**	https://github.com/younglululu/CAFE	Stand-alone program for alignment-free comparison of metagenome data
**Cancer PanorOmics**	http://panoromics.irbbarcelona.org	Mapping of cancer mutations to 3D protein–protein interaction sites
**COFACTOR**	http://zhanglab.ccmb.med.umich.edu/COFACTOR/	Structure-based protein function annotation
**compleXView**	http://xvis.genzentrum.lmu.de/compleXView	Protein-protein interaction based on affinity purification mass spectrometry
**ConTra v3**	http://bioit2.irc.ugent.be/contra/v3	Transcription factor binding sites analysis
**CPC2**	http://cpc2.cbi.pku.edu.cn	Protein coding potential of RNA transcripts
**CSPADE**	http://cspade.fimm.fi/	Chemoinformatics bioactivity assay visualization
**CSTEA**	http://comp-sysbio.org/cstea/	Analysis of time-series gene expression data on cell state transitions
**DEOGEN2**	http://deogen2.mutaframe.com/	Prediction of deleterious mutations in proteins
**DNAproDB**	http://dnaprodb.usc.edu	Structural analysis of DNA–protein complexes
**DSSR**	http://jmol.x3dna.org	DNA and RNA structure visualization
**DynOmics**	http://dyn.life.nthu.edu.tw/oENM/	Protein molecular dynamics using elastic network models
**EBISearch**	http://www.ebi.ac.uk/ebisearch	Web services text search in EMBL-EBI data
**FireProt**	http://loschmidt.chemi.muni.cz/fireprot	Design of thermostable proteins
**GalaxyHomomer**	http://galaxy.seoklab.org/cgi-bin/submit.cgi?type=HOMOMER	Prediction of protein homo-oligomer structure
**GASS-WEB**	http://gass.unifei.edu.br/	Identification of enzyme active sites
**GeMSTONE**	http://gemstone.yulab.org/	Genetic variant prioritization in human disease
**Gene ORGANizer**	http://geneorganizer.huji.ac.il	Linkage of human genes to their affected body organs
**GenProBiS**	http://genprobis.insilab.org	Mapping of SNPs to protein binding sites
**GEPIA**	http://gepia.cancer-pku.cn/	Analysis of differential gene expression in cancer
**GeSeq**	https://chlorobox.mpimp-golm.mpg.de/geseq.html	Annotation of chloroplast genomes
**GibbsCluster**	http://www.cbs.dtu.dk/services/GibbsCluster-2.0	Detection of protein short linear motifs
**GPCR-SSFE 2.0**	http://www.ssfa-7tmr.de/ssfe2/	Homology modeling of G-protein coupled receptors
**GWAB**	http://www.inetbio.org/gwab/	Network-based genome wide association analysis
**HDOCK**	http://hdock.phys.hust.edu.cn/	Protein–protein and protein–DNA/RNA docking
**HGVA**	http://bioinfodev.hpc.cam.ac.uk/web-apps/hgva	Archive of human genetic variant annotations
**HH-MOTiF**	http://chimborazo.biochem.mpg.de/	Detection of protein short linear motifs
**I-TASSER-MR**	http://zhanglab.ccmb.med.umich.edu/I-TASSER-MR/	Protein structure modeling for X-ray crystallography
**INTAA**	http://bioinfo.uochb.cas.cz/INTAA/	Analysis of amino acid interaction energies
**IntaRNA 2.0**	http://rna.informatik.uni-freiburg.de/IntaRNA/Input.jsp	Prediction of interactions between RNA molecules
**IslandViewer 4.0**	http://www.pathogenomics.sfu.ca/islandviewer4/	Prediction of bacterial genomic islands (horizontal gene transfer)
**kpLogo**	http://kplogo.wi.mit.edu/	Detection and visualization of short sequence motifs
**LigParGen**	http://jorgensenresearch.com/ligpargen	Force field parameters for molecular dynamics
**LimTox**	http://limtox.bioinfo.cnio.es	Text mining for compound toxicity
**mCSM-NA**	http://structure.bioc.cam.ac.uk/mcsm_na	Prediction of protein mutation effect on nucleic acid binding affinity
**MicrobiomeAnalyst**	http://microbiomeanalyst.ca	Analysis of microbiome data
**MinePath**	http://www.minepath.org	Differential expression analysis for regulatory network subpaths
**ModFOLD6**	http://www.reading.ac.uk/bioinf/ModFOLD/	Protein structure quality assessment
**mTCTScan**	http://jjwanglab.org/mTCTScan	Mutation prioritization for cancer drug response
**MutaGene**	https://www.ncbi.nlm.nih.gov/projects/mutagene/	Visualization and analysis of mutational profiles in cancer
**NNAlign-2.0**	http://www.cbs.dtu.dk/services/NNAlign-2.0	Detection of ligand motifs for receptor–ligand interactions
**NOREVA**	http://server.idrb.cqu.edu.cn/noreva/	Evaluation of data normalization methods for mass spectrometry based metabolomics data
**Olelo**	http://www.hpi.de/plattner/olelo	Text mining in PubMed
**OmicSeq**	http://www.omicseq.org	Search for omics data in major repositories
**P4P**	http://sing.ei.uvigo.es/p4p	Bacterial strain classification based on peptide datasets
**Pathview**	http://pathview.uncc.edu/	Visualization and annotation of metabolic pathways
**pepATTRACT**	http://bioserv.rpbs.univ-paris-diderot.fr/services/pepATTRACT	Prediction of protein–peptide docking
**PharmMapper**	http://lilab.ecust.edu.cn/pharmmapper	Drug target search using pharmacophore mapping
**PhD-SNP^*g*^**	http://snps.biofold.org/phd-snpg	Deleterious SNP classification
**PIGSPro**	http://cassandra.med.uniroma1.it/AbPrediction/web/pigs.php	Modeling of immunoglobulin variable domains
**plantiSMASH**	http://plantismash.secondarymetabolites.org	Detection of biosynthetic gene clusters in plants
**PMut**	http://mmb.irbbarcelona.org/PMut/	Prediction of disease potential for protein mutations
**Prism3**	http://prism3.magarveylab.ca/prism	Prediction of natural product structures from biosynthetic gene clusters
**ProteinsAPI**	http://www.ebi.ac.uk/proteins/api	Web service for protein data from UniProtKB
**ProteinsPlus**	http://proteins.plus	Structure-based modeling of proteins
**ProteoSign**	http://bioinformatics.med.uoc.gr/ProteoSign	Protein differential abundance analysis
**ReFOLD**	http://www.reading.ac.uk/bioinf/ReFOLD/	Protein structure refinement
**RegulatorTrail**	https://regulatortrail.bioinf.uni-sb.de	Analysis of transcription factors and target genes
**RiPPMiner**	http://www.nii.ac.in/rippminer.html	Prediction of chemical structures for ribosomally synthesized and post translationally modified peptides
**RNA workbench**	https://github.com/bgruening/galaxy-rna-workbench	Stand-alone collection of tools for analyzing RNAseq and RNA sequence data
**RNA-MoIP**	http://rnamoip.cs.mcgill.ca/	Prediction of RNA 2D and 3D structure
**SBSPKSv2**	http://www.nii.ac.in/sbspks2.html	Analysis of polyketide synthases
**SCENERY**	http://mensxmachina.org/en/software/	Network reconstruction from cytometry data
**SDM**	http://structure.bioc.cam.ac.uk/sdm2	Prediction of stability in protein mutants
**SeMPI**	http://www.pharmaceutical-bioinformatics.de/sempi/	Prediction of polyketide synthase products from biosynthetic gene clusters
**SLiMSearch**	http://slim.ucd.ie/slimsearch/	Detection of protein short linear motifs
**SODA**	http://protein.bio.unipd.it/soda/	Prediction of solubility in protein mutants
**SpartaABC**	http://spartaabc.tau.ac.il/webserver	Sequence simulation with indels
**ThreaDomEx**	http://zhanglab.ccmb.med.umich.edu/ThreaDomEx	Prediction of protein domains and domain boundaries
**Tools at EMBL-EBI**	http://www.ebi.ac.uk/Tools/webservices/	Web service tools from EMBL-EBI
**TraitRateProp**	http://traitrate.tau.ac.il/prop	Test of sequence evolution association with phenotype
**TRAPP**	http://trapp.h-its.org	Analysis of protein binding site dynamics
**VCF.Filter**	https://biomedical-sequencing.at/VCFFilter/	Stand-alone program for filtering and annotating genetic variants in vcf files
**Web3DMol**	http://web3dmol.duapp.com/	Protein structure visualization
**WebGestalt**	http://www.webgestalt.org	Gene set functional enrichment analysis
**WoPPER**	http://WoPPER.ba.itb.cnr.it/	Detection of bacterial genome regions with coordinated gene expression changes
**XSuLT**	http://structure.bioc.cam.ac.uk/xsult	Annotation and visualization of protein multiple sequence alignment

**Topics**. The papers in the Web Server Issue reflect current and emerging trends in computational biology research. Of particular note this year are several websites dealing with biosynthetic gene clusters (BGCs) in bacteria, fungi, and plants. The potentially useful secondary metabolites or natural products produced by these gene clusters include novel antibiotics, polyketides (PKs), nonribosomal peptides (NRPs), and ribosomally synthesized and posttranslationally modified peptides (RiPPs). The websites use genome mining to identify BGCs, predict the structures of the metabolites, or identify BGCs which produce metabolites with chemical structures similar to a query compound. Seven papers deal with these topics: **antiSMASH 4.0, ARTS, plantiSMASH, PRISM 3, RiPPMiner, SBSPKSv2** and **SeMPI**.

Other papers in this year's issue fall into the following broad categories: (i) gene expression and enrichment analysis, (ii) prediction of functional and structural effects of mutations, (iii) protein structure, binding, and docking, (iv) data visualizations and (v) miscellaneous topics.

The gene expression category contains eight papers, including: **agriGO v2**, an update which provides GO enrichment analysis in plants, **GEPIA**, a tool for gene expression and survival analysis in cancer data sets, **WebGestalt**, an update on a popular collection of tools for gene set functional enrichment, and **WoPPER**, which detects bacterial chromosome regions which exhibit coordinated gene expression changes.

The mutational effects category contains ten papers, including **GenProBiS**, which maps variants to protein binding sites and allows inspection of the interaction with bound ligands, **mCSM-NA**, which predicts the effect of protein mutations on nucleic acid binding affinities, and **mTCTScan**, which prioritizes mutations that will affect cancer drug response.

The protein structure and binding category contains 18 papers, including: **DynOmics**, for molecular dynamics simulations using elastic network models, **LigParGen**, which generates molecular dynamics force field parameters for organic ligands, **ModFOLD6**, an update which provides quality assessment for protein 3D models, **ReFOLD**, which refines protein 3D models, and **TRAPP**, which detects transient binding pockets that arise from internal protein motion.

The data visualization category contains seven papers, including: **BioAtlas**, for visualizing microbial taxa distributions both geographically and on the human body, **DSS**R, which adds support for RNA 3D structure, including base pairs, double helices and hairpin loops, to the JSmol/Jmol biomolecular structure visualization software, **kpLogo**, which detects ultra-short sequence motifs from a multiple alignment and shows, at each alignment position, the most significant starting *k*-mer, **MutaGene**, for visualizing cancer mutational profiles, and **Web3DMol**, for protein 3D structure visualization.

The large miscellaneous category includes: **IslandViewer 4.0**, an update which detects bacterial horizontal gene transfer, **SLiMSearch**, an update which scans proteomes for new instances of short linear motifs (SLiMs), occurring in protein disordered regions, and provides a variety of annotation and filtering options, **ProteoSign**, an application for protein differential abundance analysis using mass spectrometry data, **compleXView**, which infers protein complexes using mass spectrometrey abundance and protein contact data, **BAR 3.0**, and **COFACTOR**, both updates, which provide protein function annotation, **IntaRNA 2.0**, an update which predicts and analyzes RNA-RNA interactions, **RNA-MoIP**, for predicting RNA 2D and 3D structures, and **SpartaABC**, a sequence generation tool which incorporates indels.

**Stand-alone programs and web services**. The Web Server issue additionally has special sections for stand-alone tools for high-throughput data analysis, which must be installed on the researcher's computer, and web services for data and analysis, which are accessed programmatically rather than by manual interaction with a website. Three papers report on large collections of web services, **EBI Search Engine**, an update for text search in the EMBL-EBI data repositories, **Bioinformatics Tools from EMBL-EBI**, an update on bioinformatics analysis tools, and **Proteins API**, for protein associated data from the UniProt Knowledgebase. Three papers report on stand-alone tools, **CAFE**, for alignment-free comparison of genomes and metagenomes, **VCFFilter**, for filtering and annotating genetic variants stored in large vcf files, and **RNA Workbench**, for analyzing RNAseq and RNA sequence data.

In a quest to support innovation in the development of stand-alone tools, this year I’ve allowed submissions that are installed using the docker or conda environments. These environments allow tools with many dependencies to have all the software parts packaged together in order to simplify installation. Additionally, they aid reproducibility because earlier packages can be preserved even if the software parts themselves are updated. The major complication in using these packages is the requirement of prior installation of software to handle the environments. The goal, not easily achieved, is a ‘one-click’ installation, and it remains to be seen if researchers will choose to use these types of resources. I welcome feedback on this point. RNA Workbench, mentioned above, is in this category. Going forward, I will continue to evaluate such containerized tools on a case-by-case basis and will be looking for significant added value beyond what could be obtained using a command line program.

**Graphical abstracts**. This year, for the first time, the Web Server Issue is trying out graphical abstracts for each paper, to be included in the table of contents. A graphical abstract is a visual representation of the central finding or methodology of the paper. Although this may be a challenge for papers describing websites and computational methods, there have been some interesting submissions. More details on the requirements for graphical abstracts are available at https://academic.oup.com/nar/pages/submission_webserver and we will be posting good examples to use as guidance for next year.

**Acknowledgements**. The Web Server issue arises out of the work of many people who I would like to thank. First, there are the researchers and scientific programmers who provide us high-quality, freely available web resources and who revise and improve their manuscripts and websites under considerable time pressure. Next are the hundreds of referees who conscientiously contribute their time to reviewing and helping improve the manuscripts and websites.

**Figure 1.**
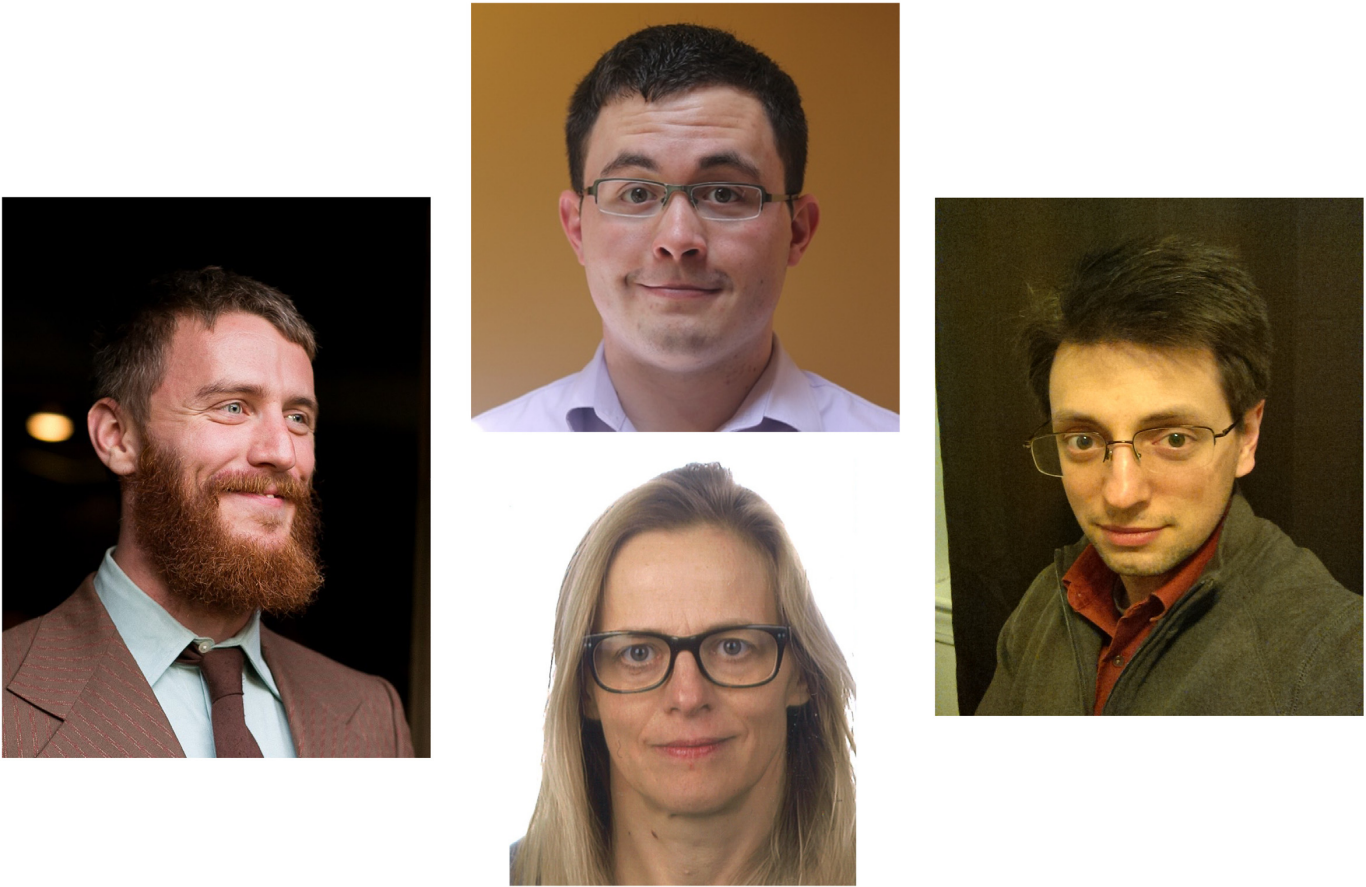
Clockwise from left: Sean Corbett, David Jenkins and Tyler Faits are PhD students in the Boston University Bioinformatics Graduate Program. Judith Mueller is a research scientist with the Rosetta Design Group, which specializes in macromolecular modeling. They provided outstanding assistance in testing the Web Server proposal websites.

My own work is made enormously easier by the dedicated editorial assistance of Fay Oppenheim, who logs the data for all the proposals and interacts with the referees, inviting, cajoling and chasing them so that their reviews are returned on time. Thank you. Thanks also to Sean Corbett, Tyler Faits and David Jenkins, PhD students in the Boston University Bioinformatics Program, and Judith Mueller, a research scientist with the Rosetta Design Group, for their hard work in carefully evaluating the proposal websites during the proposal approval phase (Figure 1). Additional thanks to Martine Bernardes-Silva, Editorial Manager, NAR, and Jennifer Boyd and the staff at Oxford University Press.

**Deadlines for 2018**. To streamline the review process, authors are required to send a one-page summary of their Web Server to the editor, Dr Gary Benson (narwbsrv@bu.edu), for pre-approval prior to manuscript submission. Authors should consult the instructions for summary submission and website design at https://academic.oup.com/nar/pages/submission_webserver. For 2018, the summary and URL address of the fully functional website must be submitted by 31 December 2017. The deadline for submission of articles is 31 January 2018.

**Instructions for submissions**. Review of a proposal includes evaluation of the summary and extensive testing of Web Server functionality. The key criteria for approval are high scientific quality, wide interest, ability to do computations on user-submitted data, and a well-designed, well-implemented, and fully functional website. Note that there is a minimum two-year interval before publication in the Web Server issue for websites, or essentially similar websites, that have been the subject of a previous publication, including in journals other than *NAR*. With respect to the website, the following are guidelines for approval.

1. It should have an easy-to-find submission page with a simple mechanism for loading test data and setting test parameters. The preferred method is one-click loading. The test data should be available for download so that users can examine the data format. Additional mechanisms that assist the user in submitting data should be implemented where appropriate, for example, automatic loading of a pdb structure file once the user has entered the appropriate identifier.
2. Output should be dynamic and rich in detail. Wherever possible, supporting evidence used in calculations and/or links to external databases containing additional information should be provided. Numerical, textual and graphical output should be mixed and visualization tools that add information or increase the user's understanding should be utilized. Note that output consisting merely of a few numerical values, a static spreadsheet, or a series of files to be opened in other programs will not be approved. Note also that for security reasons, use of FLASH and Java plugins will no longer be allowed.
3. Web servers that do not finish their calculations immediately must implement a mechanism for returning results to the user. Notification by email may be provided as an option, but an alternative that returns a web link at the time of data submission, which the user can then bookmark and access at a later time, is required. This link should ideally report the status of the job (queued, running, finished) and an estimate of the overall time for job completion. Websites that require a guest login will not be approved. Note that uploaded data and the results of analysis for each user must be private and not viewable by other users.
4. The website should be supported by an extensive help section or tutorial that guides the user through the submission process, contains details about input file formats and parameters, and explains the meaning of the output. Whenever possible, the help pages should link to dynamic output examples similar to those provided by the website. When video tutorials are used, text and figure help pages should also be available to simplify quick look-up.
5. Any proposal for a Web Server that is predictive must include details on validation of predictions from new data not used in training. *N*-fold cross validation methods will not be considered sufficient. Details should include size and composition of the validation data set (number of positive and negative cases), and several measures of predictive performance, including sensitivity, specificity and precision. Proposals are frequently rejected for lack of adequate prediction validation information.
6. Websites not clearly designed to accept and analyze user-submitted data will be rejected. This applies to those established primarily for lookup or exploration in a data set, or serve the function of ‘data aggregators.’ Authors of websites that provide novel data should consider the *NAR Database Issue* as a possible venue (see the instructions at https://academic.oup.com/nar/pages/ms_prep_database).
7. Proposals that describe a new analysis method are generally not appropriate for the Web Server issue because limited space and the rapid revision process make thorough method description and validation problematic. Authors of such methods might instead consider sending their manuscript to *NAR* as a regular computational biology paper (see the instructions for authors at https://academic.oup.com/nar/pages/Criteria_Scope#Computational%20Biology).

Gary Benson

Executive Editor

Web Server Issue

*Nucleic Acids Research*

